# Cigarette Smoking Decreases Global MicroRNA Expression in Human Alveolar Macrophages

**DOI:** 10.1371/journal.pone.0044066

**Published:** 2012-08-29

**Authors:** Joel W. Graff, Linda S. Powers, Anne M. Dickson, Jongkwang Kim, Anna C. Reisetter, Ihab H. Hassan, Karol Kremens, Thomas J. Gross, Mary E. Wilson, Martha M. Monick

**Affiliations:** 1 Department of Medicine, University of Iowa, Iowa City, Iowa, United States of America; 2 Iowa City Veteran Affairs Medical Center, Iowa City, Iowa, United States of America; 3 Department of Microbiology, University of Iowa, Iowa City, Iowa, United States of America; Pennington Biomedical Research Center, United States of America

## Abstract

Human alveolar macrophages are critical components of the innate immune system. Cigarette smoking-induced changes in alveolar macrophage gene expression are linked to reduced resistance to pulmonary infections and to the development of emphysema/COPD. We hypothesized that microRNAs (miRNAs) could control, in part, the unique messenger RNA (mRNA) expression profiles found in alveolar macrophages of cigarette smokers. Activation of macrophages with different stimuli *in vitro* leads to a diverse range of M1 (inflammatory) and M2 (anti-inflammatory) polarized phenotypes that are thought to mimic activated macrophages in distinct tissue environments. Microarray mRNA data indicated that smoking promoted an “inverse” M1 mRNA expression program, defined by decreased expression of M1-induced transcripts and increased expression of M1-repressed transcripts with few changes in M2-regulated transcripts. RT-PCR arrays identified altered expression of many miRNAs in alveolar macrophages of smokers and a decrease in global miRNA abundance. Stratification of human subjects suggested that the magnitude of the global decrease in miRNA abundance was associated with smoking history. We found that many of the miRNAs with reduced expression in alveolar macrophages of smokers were predicted to target mRNAs upregulated in alveolar macrophages of smokers. For example, miR-452 is predicted to target the transcript encoding MMP12, an important effector of smoking-related diseases. Experimental antagonism of miR-452 in differentiated monocytic cells resulted in increased expression of MMP12. The comprehensive mRNA and miRNA expression profiles described here provide insight into gene expression regulation that may underlie the adverse effects cigarette smoking has on alveolar macrophages.

## Introduction

Cigarette smoking is a prominent risk factor for many respiratory diseases including emphysema/COPD, respiratory bronchiolitis, interstitial lung disease, and desquamative interstitial pneumonitis [1], [2], [3]. In fact, a correlation exists between alveolar macrophage numbers and the severity of COPD [4], [5], [6], [7]. Aberrant gene expression in alveolar macrophages has been shown to alter the protease/anti-protease balance in the lung contributing to the development of emphysema [7], [8], [9], [10]. Of particular importance in maintaining the optimal protease/anti-protease balance is expression of matrix metalloproteinase 12 (MMP12), a macrophage secreted enzyme that degrades elastin. The importance of alveolar macrophage-derived MMP12 in emphysema is well described [8], [11].

Alveolar macrophages are essential immune effector cells in the lung with functions that include pathogen clearance and responses to inhaled environmental exposures [8], [12], [13], [14], [15], [16]. Smoking causes alveolar macrophage defects in phagocytosis, responses to pathogen-associated molecular patterns, and microbicidal activity [17], [18], [19], [20], [21]. These defects compromise alveolar macrophage-mediated protection from infectious agents [22].

Macrophage gene expression programs are altered in response to local environmental cues. These changes may underlie the regulatory role macrophages play in many disease processes [23]. The use of polarizing stimuli to activate macrophages *in vitro* identified unique macrophage gene expression programs and the associated activation phenotypes. For example, exposure to inflammatory stimuli such as IFNγ and LPS polarizes macrophages toward an M1 activated phenotype that is associated with microbicidal activity [23]. Alternatively, macrophages can be polarized to a variety of M2 phenotypes after exposure to IL-4, immune complexes, IL-10, TGFβ, or steroids [24]. Depending on the stimulus, the M2 phenotypes are associated with many activities including wound healing, immunosuppression, or production of cytokines promoting type 2 immune responses.

Transcriptional profiles of human alveolar macrophages directly isolated from nonsmokers and active smokers have shown that cigarette smoke exposure alters macrophage gene expression [1], [2], [25]. The pattern in alveolar macrophages from smokers has been suggested to reflect both suppression of M1-induced transcripts and increased expression of M2-induced transcripts [2]. The data shown here, partially replicates this finding, while proposing a new definition of the altered phenotype in smoker alveolar macrophages.

MicroRNAs (miRNAs) are small, noncoding RNAs that have an important regulatory role in gene expression programs [26], [27], [28]. Inhibition of translation and degradation of the miRNA-targeted transcripts occurs when a miRNA guides an RNA-induced silencing complex to the targeted transcript via miRNA:mRNA base pairing [29]. Each miRNA has the potential to repress the expression of hundreds of genes [30]. Expression profiling has identified miRNAs that have increased abundance in macrophages responding to inflammatory conditions [31], [32], [33], [34], [35], [36]. Despite an incomplete understanding of all transcripts targeted by the inflammation-induced miRNAs, several are known to regulate components of important signaling pathways involved in macrophage gene expression [31], [37], [38], [39], [40].

An initial study on the effect of smoking on human miRNA expression was reported by Schembri et al [41]. They identified 28 differentially expressed miRNAs when comparing primary human bronchial airway epithelium of smokers and nonsmokers. The effect of smoking on miRNA expression in human alveolar macrophages is unknown. We hypothesized that miRNAs have a role in regulating the unique gene expression program in alveolar macrophages of cigarette smokers. We examined miRNA and mRNA expression in alveolar macrophage RNA from active smokers and nonsmokers. Microarray-derived mRNA expression profiles suggested that smoking is associated with an inverse M1-type gene expression pattern in alveolar macrophages. Expression analysis of miRNAs showed a smoking dose-dependent global repression of miRNAs in alveolar macrophages. Target prediction analyses revealed many examples of downregulated miRNAs in smokers that correlated with increased expression of predicted mRNA targets. *In vitro* experiments showed a direct link between low expression of miR-452 and increased expression of MMP12 mRNA. These results are consistent with the hypothesis that miRNAs play a role in regulating gene expression in alveolar macrophages of smokers, and possibly a corresponding role in disease pathogenesis.

**Table 1:** 25 most upregulated mRNAs in alveolar macrophages of smokers.

Gene	Description	Fold Change^a^	Smoker AMs^b^	M1^c^	M2a^c^
PLA2G7	phospholipase A2, group VII	9.85	up	down	down
SPP1	secreted phosphoprotein 1 (osteopontin)	8.98	up	down	no change
CYP1B1	cytochrome P450, family 1, subfamily B, polypeptide 1	8.63	up	down	no change
ATP6V0D2	ATPase, H+ transporting, lysosomal 38 kDa, V0 subunit d2	8.41	up	ND	ND
SLC7A11	solute carrier family 7, member 11 (xCT)	6.36	up	down	down
MMP12	matrix metallopeptidase 12 (macrophage elastase)	5.83	up	up	up
FABP3	fatty acid binding protein 3	5.39	up	down	no change
RPL15	ribosomal protein L15	5.18	no change	down	no change
FLT1	fms-related tyrosine kinase 1 (VEGFR)	5.13	up	no change	no change
A2M	alpha-2-macroglobulin	4.36	up	down	no change
UCHL1	ubiquitin carboxyl-terminal esterase L1	3.79	up	no change	no change
S100B	S100 calcium binding protein B	3.53	up	no change	up
CA2	carbonic anhydrase II	3.34	up	down	0
SLC16A6	solute carrier family 16, member 6 (monocarboxylic acid transporter)	3.28	up	down	up
SSBP3	single stranded DNA binding protein 3	3.25	up	no change	no change
TDRD9	tudor domain containing 9	3.18	up	no change	no change
OR6N2	olfactory receptor, family 6, subfamily N, member 2	3.15	ND	ND	ND
HIST1H2AJ	histone cluster 1, H2aj	3.13	no change	ND	ND
C4orf18	chromosome 4 open reading frame 18 (DKFZp434L142)	3.1	up	down	no change
DNASE2B	deoxyribonuclease II beta	3.07	up	no change	no change
SDC2	syndecan 2	3.07	up	down	up
MGST1	microsomal glutathione S-transferase 1	3.03	up	up	down
AGPAT9	1-acylglycerol-3-phosphate O-acyltransferase 9	2.91	up	down	down
TM7SF4	transmembrane 7 superfamily member 4 (DCSTAMP)	2.75	up	no change	no change
LIPA	lipase A, lysosomal acid, cholesterol esterase	2.71	up	down	no change

a Change indicates smoker-to-nonsmoker expression ratio in alveolar macrophages from this study.

b Expression change of indicated mRNA described by Woodruff et al [25] in analysis of alveolar macrophage smokers and nonsmokers (GEO dataset 1269).

c Expression change of indicated mRNA described by Martinez et al [55] in analysis of monocyte-derived macrophages (MDMs) polarized toward M1 or M2a phenotypes relative to unstimulated MDMs (GEO datasets 2429 and 2430).

(“up” indicates upregulation; “down” indicates downregulation; “no change” indicates no change and “ND” indicates not determined).

**Table 2:** 25 most downregulated mRNAs in alveolar macrophages of smokers.

Gene	Description	Fold Change^a^	Smoker AMs^b^	M1^c^	M2a^c^
CXCL11	chemokine (C-X-C motif) ligand 11	−13.14	down	up	no change
CXCL9	chemokine (C-X-C motif) ligand 9	−6.59	down	up	no change
SLC19A3	solute carrier family 19 (thiamine transporter)	−5.93	down	no change	no change
EMR1	egf-like module containing, mucin-like, hormone receptor-like 1 (F4/80)	−5	down	up	no change
CXCL10	chemokine (C-X-C motif) ligand 10	−4.97	down	up	no change
PDGFD	platelet derived growth factor D	−4.65	down	no change	no change
IGF1	insulin-like growth factor 1	−4.47	down	down	no change
GBP5	guanylate binding protein 5	−4.03	down	up	no change
OVCH1	ovochymase 1	−3.86	ND	ND	ND
C8B	complement component 8, beta	−3.78	down	no change	no change
CD69	CD69 molecule	−3.61	down	no change	no change
WDR49	WD repeat domain 49	−3.32	down	ND	ND
TNFSF10	tumor necrosis factor (ligand) superfamily,member 10 (TRAIL)	−3.27	down	up	no change
IFI27	interferon, alpha-inducible protein 27 (ISG12)	−3.17	down	up	up
TRHDE	thyrotropin-releasing hormone degrading enzyme	−2.99	down	no change	no change
MYB	v-myb myeloblastosis viral oncogene homolog	−2.97	down	no change	no change
GZMA	granzyme A	−2.82	no change	up	no change
CLDN6	claudin 6	−2.67	no change	no change	no change
ARHGAP24	Rho GTPase activating protein 24	−2.64	down	no change	no change
RXFP2	relaxin/insulin-like family peptide receptor 2	−2.63	no change	ND	ND
TRPC6	transient receptor potential cation channel,subfamily C, member 6	−2.59	down	no change	no change
KLRK1	killer cell lectin-like receptor subfamily K, member 1	−2.55	no change	no change	no change
MS4A6A	membrane-spanning 4-domains, subfamily A,member 6A	−2.54	no change	down	no change
GBP3	guanylate binding protein 3	−2.52	no change	up	no change
ITIH5	inter-alpha (globulin) inhibitor H5	−2.52	down	no change	no change

a Change indicates smoker-to-nonsmoker expression ratio in alveolar macrophages from this study.

b Expression change of indicated mRNA described by Woodruff et al [25] in analysis of alveolar macrophage smokers and nonsmokers (GEO dataset 1269).

c Expression change of indicated mRNA described by Martinez et al [55] in analysis of monocyte-derived macrophages (MDMs) polarized toward M1 or M2a phenotypes relative to unstimulated MDMs (GEO datasets 2429 and 2430).

(“up” indicates upregulation; “down” indicates downregulation; “no change” indicates no change and “ND” indicates not determined).

## Materials and Methods

### Ethics Statement

All procedures and protocols described in this communication were approved by the University of Iowa Institutional Review Board. Written informed consent was obtained and all clinical investigation has been conducted according to the principles expressed in the Declaration of Helsinki.

### Alveolar Macrophage Donors

Subjects were recruited from the community by the Iowa Institute for Clinical and Translational Science (ICTS) Clinical Core via advertisements and word-of-mouth. Inclusion criteria for case subjects required at least a 10 pack-year history of smoking, while the nonsmoker control subjects were self-reported never smokers. Subjects were excluded if they had any significant co-morbid conditions such as pregnancy or other acute or chronic disease such as pre-existing asthma, interstitial lung disease or cardiovascular disease. Subjects were also excluded if a baseline spirometry revealed the forced expiratory volume in the first second was less than 60% of the predicted value based on National Health and Nutrition Examination Survey III data set.

#### Cohort 1

The first cohort of alveolar macrophage donors consisted of 4 nonsmokers and 4 active smokers with 31±14 pack-year histories. All subjects were Caucasian. The nonsmoker group had 3 males and 1 female with a mean group age of 26±9 years. The smoker group had 2 males and 2 females with a mean group age of 51±8 years.

#### Cohort 2

The second cohort included 4 nonsmokers and 4 smokers with 31±3 pack-year histories. All subjects were Caucasian except for 1 African American in the smoker group. The nonsmoker group had 2 females and 2 males with a mean group age of 31±6 years. The smoker group had 2 males and 2 females with a mean age of 47±years.

#### Cohort 3

The third cohort included 4 nonsmokers, 4 light smokers (12±2 pack-year histories), and 4 heavy smokers (33±6 pack-year histories). All subjects were Caucasian except for 1 African American in each of the smoker groups. Each of the three groups had 2 male and 2 female donors. The mean age for the groups was 35±12 years for the nonsmokers, 36±7 years for the light smokers, and 51±7 years for the heavy smokers.

### Bronchoalveolar Lavage

After informed consent was obtained, subjects underwent standard flexible bronchoscopy [42]. Local anesthesia with lidocaine instillation into the upper airway was followed by bronchoalveolar lavage whereby 20 ml of normal saline was instilled into a tertiary bronchus up to five times in three different lung segments. The first collection out of five was discarded to avoid possible contamination with upper airway secretions or lidocaine. The remaining lavage was filtered through sterile gauze and centrifuged at 200×g for 5 minutes to pellet cellular material. The resulting pellet was suspended in phosphate buffered saline (PBS) and centrifuged at 200×g for 5 minutes. A sample of the cells were labeled with Wright stain and microscopically examined to determine the proportion of the cells that were macrophages [43], [44], [45], [46]. Aliquots of 5×10^6^ cells were stored at −80°C until RNA isolation procedure was performed. Cell yields from bronchoscopy in cohort 1 averaged 25±3×10^6^ cells for the nonsmokers and 67±4×10^6^ cells for the active smokers. In all three cohorts the procedure generated a relatively pure population of alveolar macrophages with fewer than 5% neutrophils or lymphocytes in the bronchoalveolar lavage fluid.

**Figure 1:**
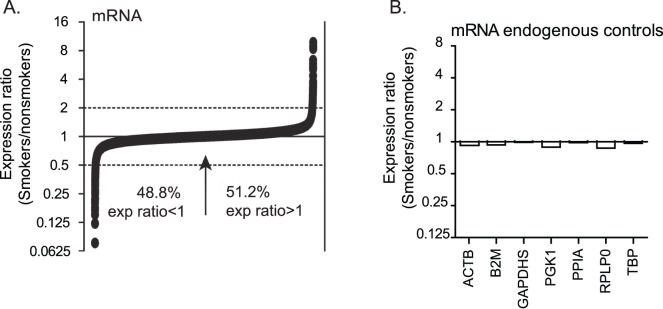
Expression profiling indicates similar numbers of mRNAs are upregulated and downreglated in alveolar macrophages of cigarette smokers and nonsmokers. Smoker-to-nonsmoker mRNA expression ratios were determined using RNA from alveolar macrophages as template in GeneChip Human Exon 1.0 ST cDNA microarrays (Affymetrix). The RNA was collected from alveolar macrophages directly isolated from four nonsmokers and four smokers (cohort 1). **A)** Smoker-to-nonsmoker expression ratios are represented by black circles in order from lowest to highest for the 17,860 detected cDNAs. The arrow indicates the point where specific mRNA expression ratios in smokers and nonsmokers = 1. **B)** The expression ratios are shown for several commonly used endogenous controls. (ACTB = actin, beta; B2M = beta-2-microglobulin; GAPDHS = glyceraldehyde-3-phosphate dehydrogenase, spermatogenic; PGK1 = phosphoglycerate kinase 1; peptidylprolyl isomerase A; RPLP0 = ribosomal protein, large, P0; TBP = TATA box-binding protein).

### RNA Isolation

RNA was isolated from alveolar macrophages or PMA-differentiated THP-1 cells using the mirVana miRNA Isolation kit (Applied Biosystems (ABI)). The quantity and quality of the RNA samples was assessed using an Experion Automated Electrophoresis Station (Bio-Rad). The RNA quality indicator was above 8 for all samples where values of greater than 8 indicate primarily intact RNA on a scale of 1–10. After preparation, RNA samples were stored at −80°C until use.

### mRNA Expression Analysis

Measurements of genome-wide macrophage mRNA expression were conducted using the GeneChip Human Exon 1.0 ST Arrays (Affymetrix). Generation of labeled cDNA, hybridizations, and scanning of the microarray were performed under contract by the University of Iowa DNA facility. The resulting data were analyzed using the Partek Genomics Suite version 6.5 (Partek). The data were assessed for quality and subjected to robust multiarray averaging (RMA) normalization. The normalized data were then analyzed using an ANOVA model with linear contrasts to calculate p-values and smoker-to-nonsmoker expression ratios. The false discovery rate (FDR) step-up method [47] was applied to correct for multiple testing. The expression data has been deposited in NCBI Geo repository (GSE34517).

### miRNA Expression Analysis

RNA from alveolar macrophages of nonsmokers and active smokers was reverse transcribed with MultiScribe Reverse Transcriptase (ABI) using Megaplex Primers version 2.0 (ABI). Changes in miRNA expression was then determined using human TaqMan Low Density Arrays version 2.0 (ABI). Ct values calculated using SDS version 2.4 (ABI) were exported to the Partek Genomics Suite to calculate smoker-to-nonsmoker expression ratios. RMA-normalized data were subjected to an ANOVA model with linear contrasts to calculate p-values.

Principle component analysis (PCA) was performed using standard functions built in MATLAB software version 7.9 (MathWorks). This analysis identified a representative miRNA within each cluster with the highest Pearson correlation between its expression profile and the first principal component from our PCA analysis [48]. Cluster analysis was performed using ΔCt values of the miRNAs with expression changes of >2-fold was accomplished using the CiMminer web interface and the default Euclidean clustering algorithm (http://discover.nci.nih.gov/cimminer/).

**Figure 2:**
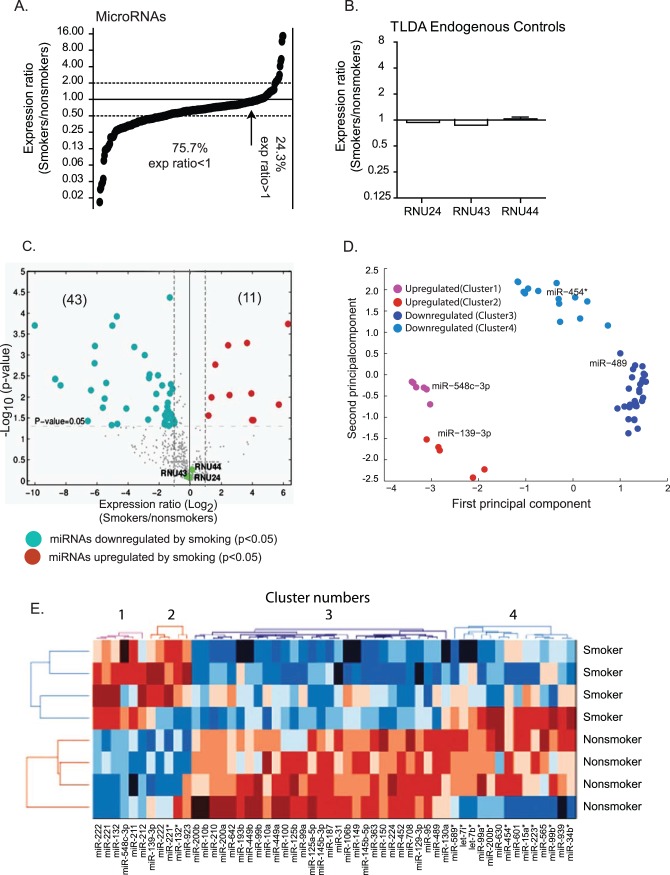
Expression profiling indicates a global repression of total miRNA abundance in alveolar macrophages of cigarette smokers. Smoker-to-nonsmoker miRNA expression ratios were determined using RNA from alveolar macrophages as template in TaqMan Low Density Array v2.0 RT-qPCR assays (ABI). The endogenous control, RNU48, was used to normalize the data. The eight RNA samples used as template in figure 1 were also used in these TLDA assays (cohort 1). **A)** Smoker-to-nonsmoker expression ratios are represented by black circles in order from lowest to highest for the 481 detected miRNAs. The arrow indicates the point where miRNA expression ratios in smokers and nonsmokers = 1. **B)** The expression ratios are shown for three additional endogenous control options provided with the TLDA assay are shown. **C)** The expression ratios and p-values of the 481 detected miRNAs are shown using a volcano plot. The significantly upregulated (red) and downregulated (blue) miRNAs are indicated along with the endogenous controls (green). **D)** The 54 miRNAs with smokers-to-nonsmokers expression ratios greater than 2 are shown following principle component analysis (PCA) with MATLAB software. This analysis identified a representative miRNA within each cluster with the highest Pearson correlation between its expression profile and the first principal component from our PCA analysis. **E)** Clustering analysis of the 54 regulated miRNAs was performed using CiMminer based on ΔCt-values of the TLDA results. The 4 clusters identified by PCA are labeled. Upregulated miRNAs are designated by various shades of red and downregulated miRNAs by various shades of blue.

### Validation of Changes in Individual miRNAs

Validation studies were performed using alveolar macrophage RNA collected from the initial array group (cohort 1) and a non-redundant sample set (cohort 2). Individual TaqMan MicroRNA Assays corresponding to assays including in the TLDA version 2.0 assays (ABI) were used to document abundance of the mature forms of three of the downregulated miRNAs. Briefly, 10 ng RNA was reversed transcribed with MultiScribe RT (ABI) using a miRNA-specific stem loop primer. Then, PCR with TaqMan Universal PCR Master Mix (ABI) was performed using miRNA-specific real time primers. Expression levels were defined as a ratio between the Ct values of the indicated miRNA and the endogenous control, RNU48.

### Analysis of miRNA Target Prediction Expression

Using the Partek Genomics Suite software, each indicated miRNA was used to query the TargetScan [49] and MicroCosm [50] databases to identify predicted targets. The smoker-to-nonsmoker expression ratio and ANOVA-derived p-value of each predicted miRNA target were exported from the GeneChip Human Exon microarray results to an Excel spreadsheet (Microsoft). Excel was used to filter data by expression ratios and p-values.

### Expression of Predicted Targets in miRNA Antagonist-transfected THP-1 Cells

THP-1 cells were maintained in RPMI 1640 (Gibco) supplemented with fetal bovine serum (10%; Gibco). Cells were incubated with PMA (5 ng/ml; Sigma-Aldrich) for 18 hours to induce differentiation toward a macrophage phenotype [51]. They were then transfected with the miR-452 or negative control mirVana miRNA Inhibitors (25 nM; ABI) using RNAiMAX (Invitrogen). RNA was purified from cell lysates collected at 24 hours post-transfection. Total RNA (300 ng) was reverse-transcribed to cDNA using iScript cDNA Synthesis kit (Bio-Rad). SYBR Green-based quantitative PCR reactions (BioRad) were performed as previously described [52]. Specificity of the amplification was confirmed using melting curve analysis. Expression levels were defined as a ratio between the threshold cycle (Ct) values of MMP12 or TM7SF4 and the endogenous control, HPRT. The primers (Integrated DNA Technologies) used in the PCR reactions were: MMP12, forward 5′-aggtggaatcctagcccatgcttt-3′, reverse 5′-tcaggatttggcaagcgttggttc-3′; TM7SF4, forward 5′-tgggagtttgctgtttggttgctc-3′, reverse 5′-atcaaagcattcctgccttcacgc-3′; HPRT, forward 5′-ccatcacattgtagccctctgtgt-3′, reverse 5′-actgcctgaccaaggaaagcaaag-3′.

**Table 3:** miRNAs upregulated >2-fold in alveolar macrophages of smokers.

miRNA	miRNA Family	Polycistronic miRNA Precursor	Fold Change	p-value
miR-132*	miR-132	miR-132/212	78.47	0.0002
miR-139-3p	N/A	N/A	52	0.0151
miR-548c-3p	miR-548	miR-548c/548z	16.71	0.0357
miR-211	miR-204	N/A	15.77	0.0355
miR-222*	miR-221	miR-221/222	15.3	0.0082
miR-221	miR-221	miR-221/222	12.59	0.0005
miR-221*	miR-221	miR-221/222	5.77	0.009
miR-222	miR-221	miR-221/222	5.42	0.0006
miR-132	miR-132	miR-132/212	3.05	0.0017
miR-212	miR-132	miR-132/212	2.58	0.0102

**Table 4:** miRNAs downregulated >2-fold in alveolar macrophages of smokers.

miRNA	miRNA Family^a^	Polycistronic miRNA Precursor^b^	Fold Change^c^	p-value
miR-452	miR-452	miR-224/miR-452	−1030.3	<0.001
miR-129-3p	N/A	N/A	−414.23	0.004
miR-31	N/A	N/A	−326.38	0.005
miR-34b*	miR-34	miR-34b/miR-34c	−96.89	0.037
miR-210	N/A	N/A	−84.03	0.007
miR-200b*	miR-8	miR-200a/miR-200b/miR-429	−71.68	0.002
miR-150	N/A	N/A	−70.28	0.001
miR-449a	miR-449	miR-449a/miR-449b/miR-449c	−49.46	0.018
miR-200a	miR-8	miR-200a/miR-200b/miR-429	−45.64	0.011
miR-10b	miR-10	N/A	−42.63	0.005
miR-449b	miR-449	miR-449a/miR-449b/miR-449c	−33.37	0.047
miR-224	miR-452	miR-224/miR-452	−32.41	<0.001
miR-708	N/A	N/A	−26.52	<0.001
let-7b*	let-7	let-7a-3/let-7b/miR-4763	−25.9	0.044
miR-149	N/A	N/A	−17.34	0.019
miR-187	N/A	N/A	−12.18	0.001
miR-125a-5p	miR-125	let-7e/miR-99b/miR-125a	−7.64	0.001
miR-130*	miR-130	N/A	−6.56	0.035
miR-363	miR-363	miR-106a-92 cluster	−6.3	0.004
miR-99a*	miR-99	let-7c/miR-99a	−6.15	0.003
miR-99b	miR-99	let-7e/miR-99b/miR-125a	−4.6	0.019
miR-10a	miR-10	N/A	−4.55	0.008
miR-95	miR-95	N/A	−4.23	0.003
miR-489	N/A	miR-489/miR-653	−3.5	0.005
miR-429	miR-8	miR-200a/miR-200b/miR-429	−3.36	0.05
miR-939	N/A	miR-1234	−3.15	0.045
miR-642	miR-642	N/A	−3.11	0.04
miR-99b*	miR-99	let-7e/miR-99b/miR-125a	−3.1	0.027
miR-200b	miR-8	miR-200a/miR-200b/miR-429	−2.94	0.03
let-7i*	let-7	N/A	−2.87	0.023
miR-99a	miR-99	let-7c/miR-99a	−2.78	0.017
miR-106b	miR-17	miR-25/mir-93/miR-106b	−2.78	0.033
miR-100	miR-99	let-7a-2/miR-100	−2.73	0.014
miR-589	N/A	N/A	−2.65	0.022
miR-15a*	miR-15	miR-15a/miR-16-1	−2.61	0.049
miR-146b-3p	miR-146	N/A	−2.49	<0.001
miR-125b	miR-125	N/A	−2.48	0.008
miR-601	N/A	N/A	−2.38	0.005
miR-630	N/A	N/A	−2.34	0.025
miR-146b-5p	miR-146	N/A	−2.2	0.006
miR-223*	N/A	N/A	−2.18	0.037
miR-454*	N/A	N/A	−2.14	0.034
miR-193b	miR-193	miR-193b/miR-365a	−2.07	0.043

a Family names as specified by miRBase release 18.

b Clustered miRNAs described in miRBase release 18 were assumed to be polycistronic pri-miRNAs.

c Fold change indicates miRNA expression in alveolar macrophages of smokers compared to nonsmokers.

## Results

### Alveolar Macrophages from Smokers Displayed an “Inverse” M1 Gene Expression Profile

Cigarette smoking has been shown to alter the transcriptional profile of human alveolar macrophages in a consistent manner as reported by two independent groups [1], [2], [25]. Prior to measuring miRNA expression in alveolar macrophages from four normal healthy subjects and four cigarette smokers (cohort 1), microarrays were performed to compare the transcriptional profile of these donors to previous reports.

Tables 1 and 2 display the most upregulated and downregulated mRNAs, respectively, with p-values <0.05 in alveolar macrophages from smokers compared to nonsmokers. These regulated genes were compared to an independent mRNA expression profiling study in alveolar macrophages from 15 smokers and 15 nonsmokers by Woodruff et al., summarized in the 4^th^ column of Tables 1 and 2 ([25] and GEO dataset GDS1269). Results from our study were in strong agreement with this previous study. Most of the highly regulated mRNAs were similarly regulated in both studies and none of these mRNAs were regulated in the opposite direction (compare 3^rd^ and 4^th^ columns within Tables 1 and 2).

To further evaluate the extent to which the gene expression profile in alveolar macrophages from smokers contrasts with an M1 profile, the highly regulated mRNAs listed in Tables 1 and 2 were compared to results reported by Martinez et al [53] that described the transcriptional profile of unstimulated, M1-polarized (IFNγ- and LPS-treated), and M2a-polarized (IL-4 treated) macrophages (GEO datasets GDS2429 and GDS2430). The alveolar macrophages from smokers have an “inverse” M1 gene expression profile because not only were mRNAs that are upregulated in M1 macrophages downregulated in alveolar macrophages from smokers, similar to the observations of Shaykhiev et al [2], but also mRNAs downregulated by M1 polarization were often upregulated in smoker alveolar macrophages (compare 3^rd^ and 5^th^ columns within Tables 1 and 2). Unlike the M1-regulated mRNAs, there was not any discernible correlation between mRNAs regulated in alveolar macrophages in response to cigarette smoking and mRNAs regulated in macrophages in response to *in vitro* M2a-polarizing conditions.

**Figure 3:**
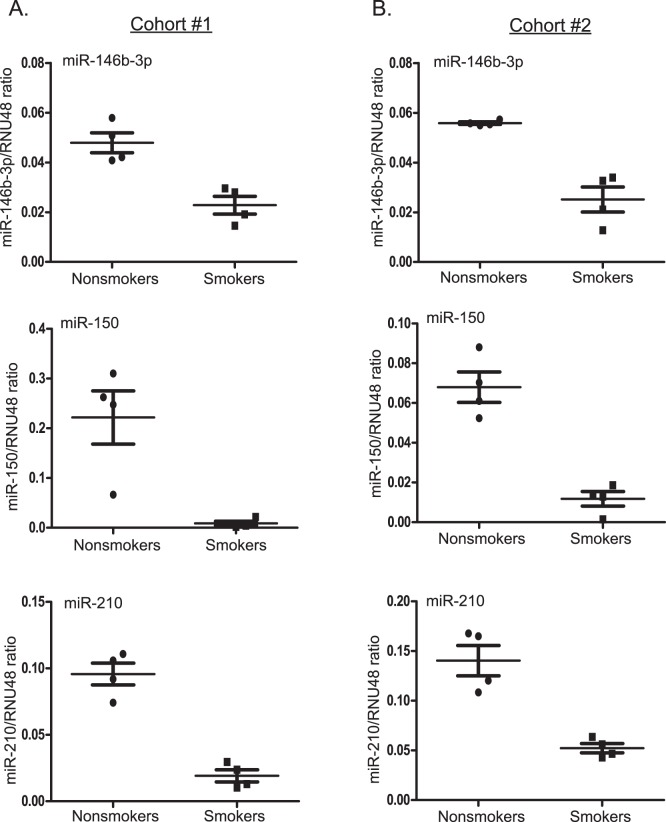
Expression profiling results were validated for select miRNAs in samples from the original alveolar macrophage donors and additional, non-redundant donors. Individual miRNA qRT-PCR expression assays were used to evaluate the expression of miR-146b-3p, miR-150, and miR-210. Expression of these miRNAs was determined using **A)** RNA analyzed previously in the TLDA assays (cohort 1) and **B)** RNA obtained from an independent set of donors (cohort 2). The mean expression with SEM of each miRNA is shown as a ratio to RNU48 for each sample.

A global mRNA abundance increase was reported in lung tissue from a rat model of cigarette smoking [54]. Among the mRNAs with p-values <0.05, more transcripts were upregulated in smoker alveolar macrophages relative to nonsmoker alveolar macrophages when using a 2-fold change cut-off. Specifically, 70 mRNAs were upregulated and 48 mRNAs were downregulated. However, when assessing all mRNAs detected by the microarrays, there were approximately equal numbers of mRNAs with smoker-to-nonsmoker expression ratios greater than 1 (51.2%) and less than 1 (48.8%) in the alveolar macrophage samples (Figure 1A) and the expression ratios of mRNAs commonly used as endogenous controls were each close to 1 (Figure 1B). While there were significant differences in expression of specific mRNAs, there was no apparent global shift in global mRNA levels in human alveolar macrophages of smokers and nonsmokers.

### Total miRNA Abundance is Reduced in Smoker Alveolar Macrophages

To address our hypothesis that miRNAs have a role in maintaining the unique smoking-associated gene expression profile, we next obtained miRNA expression profiles. TaqMan Low Density Array (TLDA) assays were used to measure miRNA expression in the same RNA samples from alveolar macrophages of smokers and nonsmokers that had been used to analyze mRNA expression (cohort 1). TLDA assays use quantitative RT-PCR to specifically measure the abundance of 667 mature human miRNAs. The total miRNA abundance appeared to be lower in alveolar macrophages from cigarette smokers compared to nonsmokers, with a median expression ratio of 0.63. Therefore, the majority (75.7%) of miRNA smoker-to-nonsmoker expression ratios was less than 1 (Fig 2A). Interestingly, the general repression of miRNA expression in alveolar macrophages of cigarette smokers coincides with reports that cigarette smoke reduces overall miRNA expression in primary human epithelial cells [41] and in lung tissues of rats [54].

The expression of miRNAs was determined using a small nucleolar RNA, RNU48, as an endogenous control. Three other small nucleolar RNAs (RNU24, RNU43, and RNU44) were included in the TLDA assays as alternative endogenous controls. The expression of all three of the additional controls was unchanged when calculated using RNU48 as an endogenous control (Fig 2B). The general repression of global miRNAs described in figure 2A is unlikely to be caused by an artifactual skewing of the data due to unreliable endogenous controls since all four endogenous controls available on the TLDA assays were in agreement with each other.

**Figure 4:**
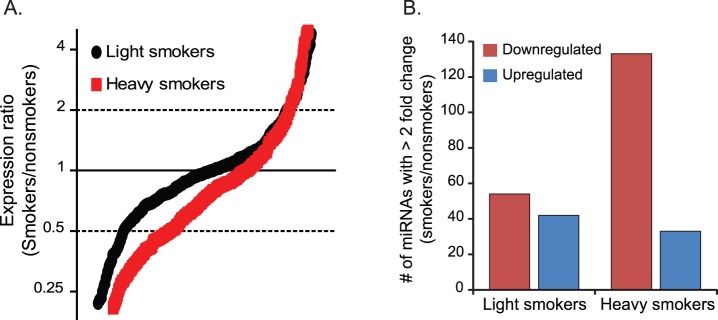
Expression profiling of a second data set indicates a global repression of total miRNA abundance in alveolar macrophages of cigarette smokers. Nonsmoker, light smoker, and heavy smoker miRNA expression ratios were determined by TLDA assays using RNA from alveolar macrophages (cohort 3). The endogenous control, RNU48, was used to normalize the data. **A)** Smoker-to-nonsmoker expression ratios are represented by black circles (light smokers) and red circles (heavy smokers) in order from lowest to highest for 277 and 281 detected miRNAs, respectively. **B)** The number of miRNAs with a greater than 2-fold change between the two smoker groups and the nonsmokers are displayed.

The overall repression of miRNA expression in alveolar macrophages of smokers compared to nonsmokers was also observed when comparing the number of miRNAs with statistically significant (p<0.05) abundance differences. For example, when using a 2-fold cut-off, 11 miRNAs have increased abundance and 43 miRNAs have decreased abundance (Fig 2C). General information for the upregulated and downregulated miRNAs is shown in Table 3 and Table 4, respectively. We list 10 miRNAs in Table 3, rather than the 11 in the data set because miR-923 is no longer considered a miRNA by miRBase. Principal component analysis (PCA) groups these 54 miRNAs into four clusters (Clusters 1–4) with the upregulated miRNAs and the downregulated miRNAs each separated into two clusters (Fig 3D). The PCA analysis identified a representative miRNA (miR-548c-3p, miR-139-3p, miR-489, and miR-454*) for each of the four clusters. Although the significance is unclear, we note that clusters 2 and 4 are dominated by passenger strands while clusters 1 and 3 are dominated by guide miRNA strands (Fig 3E). miRNA passenger strands are the strand of each miRNA-duplex that is less likely to be loaded into the RNA-induced silencing complex (RISC) [52], [53].

**Table 5:** Downregulated miRNAs in alveolar macrophages of smokers and corresponding predicted mRNA targets with upregulated expression.

miRNA	Predicted mRNA target^a^
miR-95	PRSS21 (M)
miR-99a	UGCG (M)
miR-99b	UGCG (M)
miR-100	UGCG (M)
miR-106b	C10orf58 (M)
miR-125b	ATP13A3 (T), BCAT1 (T), C10orf58 (T), CSF (M), LIPA (T), TXNRD1 (T)
miR-130a	ATP13A3 (T), BCAT1 (T)
miR-187	FAIM (M), MGST (M), UCHL1 (M)
miR-193b	GCLC (M), SLC16A6 (M & T)
miR-200b	CCDC102B (M), CYP1B1 (T), FLT1 (T)
miR-363	SLC16A1
miR-429	ATP13A3 (M), CCDC102B (M), CSF1 (M), CYP1B1 (T), FLT1 (T), SDC2 (T)
miR-449a	C15orf48 (M), FABP3 (M), PRSS21 (M)
miR-449b	C15orf48 (M), FABP3 (M)
miR-452	CCDC102B (M), GSR (M), **MMP12 (M),** MOSPD1 (M), SPRY2 (M), TDRD9 (M), **TM7SF4 (M),** C10orf58 (M), SLC7A11 (T)
miR-601	CA2 (M)
miR-630	C15orf48 (M), CA2 (M), PLA2G7 (M), SLC04C1 (T), SPRY2 (M)
miR-708	CSF1 (T)

a Gene symbols of putative miRNA targets with the prediction algorithm indicated within parenthesis (“M” = MicroCosm; “T” = TargetScan). Text in bold identifies genes tested in correlation assays (see Figure 5).

Individual RT-PCR miRNA assays were used to confirm the observations from the TLDA assays. Three differentially expressed miRNAs (miR-146b-3p, miR-150, and miR-210) were validated using the same RNA samples from cohort 1 analyzed in the TLDA assays (Fig 3A). Furthermore, these miRNAs were similarly regulated in a second, non-redundant set of alveolar macrophage samples (cohort 2) from nonsmokers and smokers (Fig 3B).

**Figure 5:**
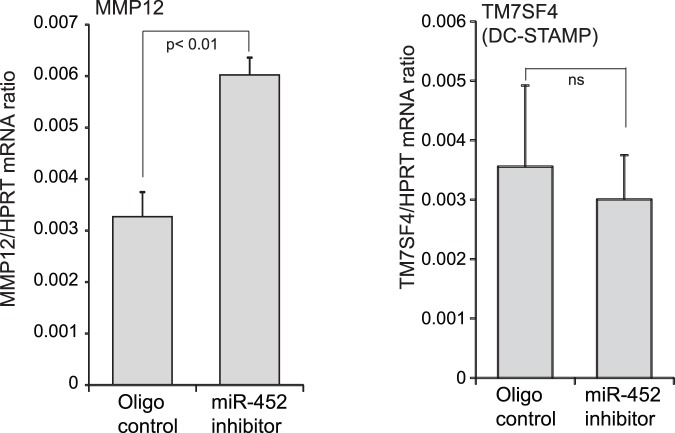
Inhibition of miRNA-452 results in increased MMP12 expression. RNA was collected from PMA-differentiated THP1 cells at 24 hours post-transfection with either a control miRNA inhibitor or a miR-452 inhibitor. Quantitative RT-PCR was used to determine the expression of putative miR-452 target. The mean expression with SEM from three independent experiments is shown for MMP12 and TM7SF4 as a ratio to HPRT for each sample.

### Decreased Global miRNA Expression is Associated with Heavy Smoking Histories

TLDA assays were used to evaluate miRNA expression profiles in a third set of alveolar macrophages (cohort 3). Four donors each were categorized as nonsmokers (no smoking history), light smokers, and heavy smokers. Light smoking resulted in a modest reduction in miRNA expression compared to nonsmokers with 54.2% of miRNAs have an expression ratio less than 1 relative to nonsmokers. The decrease in global miRNA expression was more pronounced in heavy smokers with expression ratios of less than 1 for 72.6% of the miRNAs (Figure 4A). The general downregulation of miRNAs in alveolar macrophages of smokers was also apparent by assessing the number of miRNAs using a 2-fold expression change cut-off (Figure 4B). These results from cohort 3 confirmed that smoking represses global miRNA expression and suggests that the amplitude of overall repression is directly related to the extent of an individuals’ smoking history.

### Inverse Correlations between Regulated miRNAs and the Expression of their Predicted mRNA Targets

Most mRNAs targeted for repression by miRNAs are degraded [28]. Therefore, using the results from cohort 1, the miRNAs with regulated expression were analyzed further to determine whether the expression of these miRNAs inversely correlated with the mRNA expression of their predicted targets. Using Partek Genomics Suite software, all targets predicted by either the MicroCosm or TargetScan algorithms for each miRNA were identified. Analysis of the expression for all predicted targets of each miRNA as calculated in the microarray experiments failed to reveal any examples in which a downregulated miRNA correlated with a global increase in its predicted targets, or conversely an upregulated miRNA correlated with a global decrease in its predicted targets (data not shown). There were, however, many instances in which downregulated miRNAs correlated with increased expression of a subset of predicted targets (Table 5). There was only one upregulated miRNA family, miR-221, that was associated with a downregulated predicted target, IGF1.

To test our hypothesis that miRNAs influence the mRNA expression profiles in alveolar macrophages of cigarette smokers, we evaluated whether antagonizing the function of a specific miRNA would lead to increased mRNA expression of the predicted target. We were particularly interested in whether the highly downregulated miRNA, miR-452, influenced the expression of MMP12, a protease relevant to smoking-related diseases that is highly upregulated in alveolar macrophages of smokers. Transfecting in an inhibitor of miR-452 resulted in elevated expression of MMP12 transcripts, but had no effect on another predicted target of miR-452, TM7SF4 (Figure 5).

## Discussion

This study reports on miRNA and mRNA expression in alveolar macrophages from nonsmokers and active cigarette smokers. Significant differences in both miRNA and mRNA expression were found in alveolar macrophages obtained from nonsmokers and smokers. We identified a smoking history-dependent decrease in global miRNA abundance. Importantly, we describe many examples of inverse relationships between miRNAs and their predicted mRNA targets and used an *in vitro* system to support our hypothesis that miRNAs influence the expression of an important macrophage product.

*In vitro* polarization of monocyte-derived macrophages (MDMs) leads to distinct phenotypes that have been categorized as M1, M2a, M2b, and M2c [24], [55]. This classification system is useful, particularly in defining gene expression programs related to specific polarized phenotypes. However, the extent to which these phenotypes accurately depict macrophage phenotypes *in vivo* has been difficult to determine, partly because purification of human macrophages from the tissues in which they are embedded is usually not possible. Human alveolar macrophages are unique in this aspect since relatively pure populations can be obtained from bronchoalveolar lavage fluid. A consistent alteration in gene expression profiles of human alveolar macrophages from cigarette smokers compared to nonsmokers has been reported by two independent groups of investigators [1], [2], [25]. Despite a relatively small alveolar macrophage sample size, we were able to confirm a similar transcriptional expression profile with Woodruff et al [25]. The gene expression profile of smoker alveolar macrophages was recently described as a “deactivated M1 polarization program” [2]. We suggest that the gene expression program could best be described as an “inverse” M1 profile, because M1-induced mRNAs are less abundant and M1-repressed mRNAs were more abundant in alveolar macrophages of smokers relative to nonsmokers.

Since cigarette smoking is associated with increased risk of pulmonary infections [56], understanding the mechanisms causing the inverse M1 phenotype in smoker alveolar macrophages might provide therapeutic targets for improving antimicrobial activity. Generation of M1-polarized MDMs *in vitro* is accomplished by treatment with two stimuli, typically IFNγ and either TNFα or another molecule that promotes TNFα production such as LPS [23], [55]. One possibility is that the inverse M1 phenotype described for smoker alveolar macrophages is due to a cigarette smoke-induced defect in TNFα- and/or IFNγ-induced signaling such as the NF-κB and JAK-STAT pathways. Indeed, impairment in IFNγ signaling has been described in alveolar macrophages and epithelial cells of cigarette smokers [57], [58]. The mechanism responsible for impaired IFNγ signaling is unknown. Whether miRNAs play a role promoting the inverse M1 gene expression program is currently being investigated.

We hypothesized that miRNAs are at least partly responsible for regulating the unique gene expression profile in alveolar macrophages of smokers. The RNA samples collected from cohort 1 for mRNA expression profiling of smoker and nonsmoker alveolar macrophages were also used in TLDA miRNA profile assays to compare the abundance of 667 human miRNAs. This global approach to evaluating miRNA expression identified 54 miRNAs with significantly altered expression. Among the 10 miRNAs that were significantly upregulated by >2-fold, 7 are located on two polycistronic pri-miRNAs, the miR-132/212 cluster and the miR-221/222 cluster. Therefore, increased transcription of these two polycistronic pri-miRNAs could explain almost all of the highly upregulated miRNAs in smoker alveolar macrophages. Similarly, transcriptional regulation may also be important for many of the significantly repressed miRNAs in smoker alveolar macrophages because ten downregulated miRNAs were processed from four polycistronic pri-miRNAs (miR-224/miR452, miR-200a/miR-200b/miR-429, miR-449a/miR-449b/miR-449c, and let-7e/miR-99b/miR-125a).

An important observation from the miRNA profiling experiments was that the majority of miRNAs with altered expression in alveolar macrophages of smokers compared to nonsmokers were downregulated. Functionally, this should lead to increased mRNA expression in targeted genes. Using both the TargetScan and MicroCosm target prediction algorithms to analyze the results from cohort 1, we found that 30 of the 70 statistically significant mRNAs upregulated by >2-fold were putative targets of miRNAs that were significantly downregulated by >2-fold. The most strongly downregulated miRNA in smokers, miR-452, had the most predicted targets with upregulated expression. In experiments utilizing a miR-452 inhibitor, we show that inhibition of this miRNA in differentiated THP-1 cells resulted in elevated MMP12 transcript expression.

It is likely that miRNAs are not the only mechanism that could contribute to the inverse M1 alveolar macrophage phenotype. For example, soluble TNF receptor type II is increased in the sputum of smokers with COPD [59] and in mouse models [60]. This likely sequesters TNFα and dampens the effects of M1 polarizing stimuli. Furthermore, the transcript SPP1 (encoding osteopontin), is consistently highly upregulated in studies of smoker alveolar macrophages [1], [2], [25]. Osteopontin treatment of macrophages results in proteasome-mediated degradation of STAT1 [61], [62].

There was a striking ∼50–60% reduction in total miRNA abundance in alveolar macrophages of smokers relative to nonsmokers. Global repression of miRNA expression that was also noted using non-TLDA miRNA profiling platforms in epithelial cells of smokers and in lung tissue of rats in a model of cigarette smoking [41], [54]. Furthermore, miRNA expression profiling in induced sputum of cigarette smokers and nonsmokers showed that the majority of differentially expressed miRNAs were downregulated [59], although the method of data analysis in this latter study prevented the authors from commenting on differences in global miRNA abundance between the two alveolar macrophage sample types.

The reduced global expression of miRNAs reported here in alveolar macrophages of smokers has also been reported for many cancers. For example, total miRNA abundance is lower in tumors and tumor-derived cell lines relative to corresponding normal tissue [63], [64]. The initial studies of global miRNA repression in cancer noted that mRNAs encoding the miRNA processing machinery were not altered in cancer cells [64]. Likewise, we detected no statistically significant changes in alveolar macrophages of smokers for transcripts encoding Dicer, Drosha, Ago1–4, DGCR8, TRBP, PACT, exportin-5, or GW182 (data not shown). The repression of miRNAs in cancer appears to be due to inefficient processing of primary miRNA transcripts [65]. The cigarette smoke-induced mechanism of global miRNA repression in alveolar macrophages may be due to a deficiency in miRNA processing as described for cancer, an enhancement of miRNA degradation, or changes in primary miRNA transcription. Studies are ongoing to address each of these possibilities.

The impact that global miRNA expression levels has on cancer progression was addressed by experimentally knocking down the expression of Drosha, DCGR8, and Dicer [66]. The resulting global repression of miRNA abundance promoted cellular transformation and tumorigenesis. It is tempting to speculate that the global decrease in miRNA abundance described here is a previously unrecognized link between cigarette smoking and lung cancer. It will be important to determine whether global miRNA expression is restored in former cigarette smokers.

In summary, this is the first study to analyze miRNA expression in human alveolar macrophages from nonsmokers and active smokers. The data show a global repression of miRNA levels in smokers. In addition, many of the downregulated miRNAs are predicted to target mRNAs that had increased expression in alveolar macrophages of smokers. This supports a role for miRNA expression in regulating disease-relevant changes in gene expression in smoker alveolar macrophages.

## References

[1] Heguy A, O’Connor TP, Luettich K, Worgall S, Cieciuch A, et al (2006) Gene expression profiling of human alveolar macrophages of phenotypically normal smokers and nonsmokers reveals a previously unrecognized subset of genes modulated by cigarette smoking. J Mol Med 84: 318–328. 16520944 10.1007/s00109-005-0008-2

[2] Shaykhiev R, Krause A, Salit J, Strulovici-Barel Y, Harvey BG, et al (2009) Smoking-dependent reprogramming of alveolar macrophage polarization: implication for pathogenesis of chronic obstructive pulmonary disease. J Immunol 183: 2867–2883. 19635926 10.4049/jimmunol.0900473 PMC2873685

[3] Vlahos R, Bozinovski S, Jones JE, Powell J, Gras J, et al (2006) Differential protease, innate immunity, and NF-kappaB induction profiles during lung inflammation induced by subchronic cigarette smoke exposure in mice. Am J Physiol Lung Cell Mol Physiol 290: L931–945. 16361358 10.1152/ajplung.00201.2005

[4] Hogg JC, Chu F, Utokaparch S, Woods R, Elliott WM, et al (2004) The nature of small-airway obstruction in chronic obstructive pulmonary disease. N Engl J Med 350: 2645–2653. 15215480 10.1056/NEJMoa032158

[5] Finkelstein R, Fraser RS, Ghezzo H, Cosio MG (1995) Alveolar inflammation and its relation to emphysema in smokers. Am J Respir Crit Care Med 152: 1666–1672. 7582312 10.1164/ajrccm.152.5.7582312

[6] Barnes PJ, Shapiro SD, Pauwels RA (2003) Chronic obstructive pulmonary disease: molecular and cellular mechanisms. Eur Respir J 22: 672–688. 14582923 10.1183/09031936.03.00040703

[7] Shapiro SD (1999) The macrophage in chronic obstructive pulmonary disease. Am J Respir Crit Care Med 160: S29–32. 10556166 10.1164/ajrccm.160.supplement_1.9

[8] Hautamaki RD, Kobayashi DK, Senior RM, Shapiro SD (1997) Requirement for macrophage elastase for cigarette smoke-induced emphysema in mice. Science 277: 2002–2004. 9302297 10.1126/science.277.5334.2002

[9] Shapiro SD (2005) COPD unwound. N Engl J Med 352: 2016–2019. 15888704 10.1056/NEJMe058044

[10] Shapiro SD, Ingenito EP (2005) The Pathogenesis of Chronic Obstructive Pulmonary Disease: Advances in the Past 100 Years. Am J Respir Cell Mol Biol 32: 367–372. 15837726 10.1165/rcmb.F296

[11] Hunninghake GM, Cho MH, Tesfaigzi Y, Soto-Quiros ME, Avila L, et al (2009) MMP12, lung function, and COPD in high-risk populations. N Engl J Med 361: 2599–2608. 20018959 10.1056/NEJMoa0904006 PMC2904064

[12] Bitterman PB, Rennard SI, Hunninghake GW, Crystal RG (1982) Human alveolar macrophage growth factor for fibroblasts. Regulation and partial characterization. J Clin Invest 70: 806–822. 7119116 10.1172/JCI110677 PMC370289

[13] Bitterman PB, Wewers MD, Rennard SI, Adelberg S, Crystal RG (1986) Modulation of alveolar macrophage-driven fibroblast proliferation by alternative macrophage mediators. J Clin Invest 77: 700–708. 3081573 10.1172/JCI112364 PMC423453

[14] Houghton AM, Quintero PA, Perkins DL, Kobayashi DK, Kelley DG, et al (2006) Elastin fragments drive disease progression in a murine model of emphysema. J Clin Invest 116: 753–759. 16470245 10.1172/JCI25617 PMC1361346

[15] Venet A, Hance AJ, Saltini C, Robinson BW, Crystal RG (1985) Enhanced alveolar macrophage-mediated antigen-induced T-lymphocyte proliferation in sarcoidosis. J Clin Invest 75: 293–301. 3871200 10.1172/JCI111688 PMC423439

[16] Wert SE, Yoshida M, LeVine AM, Ikegami M, Jones T, et al (2000) Increased metalloproteinase activity, oxidant production, and emphysema in surfactant protein D gene-inactivated mice. Proc Natl Acad Sci U S A 97: 5972–5977. 10801980 10.1073/pnas.100448997 PMC18543

[17] Berenson CS, Garlipp MA, Grove LJ, Maloney J, Sethi S (2006) Impaired phagocytosis of nontypeable Haemophilus influenzae by human alveolar macrophages in chronic obstructive pulmonary disease. J Infect Dis 194: 1375–1384. 17054066 10.1086/508428

[18] Chen H, Cowan MJ, Hasday JD, Vogel SN, Medvedev AE (2007) Tobacco smoking inhibits expression of proinflammatory cytokines and activation of IL-1R-associated kinase, p38, and NF-kappaB in alveolar macrophages stimulated with TLR2 and TLR4 agonists. J Immunol 179: 6097–6106. 17947684 10.4049/jimmunol.179.9.6097

[19] Hodge S, Hodge G, Ahern J, Jersmann H, Holmes M, et al (2007) Smoking alters alveolar macrophage recognition and phagocytic ability: implications in chronic obstructive pulmonary disease. American journal of respiratory cell and molecular biology 37: 748–755. 17630319 10.1165/rcmb.2007-0025OC

[20] King TE Jr, Savici D, Campbell PA (1988) Phagocytosis and killing of Listeria monocytogenes by alveolar macrophages: smokers versus nonsmokers. J Infect Dis 158: 1309–1316. 3143765 10.1093/infdis/158.6.1309

[21] Russell RE, Thorley A, Culpitt SV, Dodd S, Donnelly LE, et al (2002) Alveolar macrophage-mediated elastolysis: roles of matrix metalloproteinases, cysteine, and serine proteases. Am J Physiol Lung Cell Mol Physiol 283: L867–873. 12225964 10.1152/ajplung.00020.2002

[22] Stampfli MR, Anderson GP (2009) How cigarette smoke skews immune responses to promote infection, lung disease and cancer. Nat Rev Immunol 9: 377–384. 19330016 10.1038/nri2530

[23] Mosser DM, Edwards JP (2008) Exploring the full spectrum of macrophage activation. Nature Rev Immunol 8: 958–969. 19029990 10.1038/nri2448 PMC2724991

[24] Mantovani A, Sozzani S, Locati M, Allavena P, Sica A (2002) Macrophage polarization: tumor-associated macrophages as a paradigm for polarized M2 mononuclear phagocytes. Trends Immunol 23: 549–555. 12401408 10.1016/s1471-4906(02)02302-5

[25] Woodruff PG, Koth LL, Yang YH, Rodriguez MW, Favoreto S, et al (2005) A distinctive alveolar macrophage activation state induced by cigarette smoking. Am J Respir Crit Care Med 172: 1383–1392. 16166618 10.1164/rccm.200505-686OC PMC2718436

[26] Bartel DP (2009) MicroRNAs: target recognition and regulatory functions. Cell 136: 215–233. 19167326 10.1016/j.cell.2009.01.002 PMC3794896

[27] Ventura A, Jacks T (2009) MicroRNAs and cancer: short RNAs go a long way. Cell 136: 586–591. 19239879 10.1016/j.cell.2009.02.005 PMC3910108

[28] Guo H, Ingolia NT, Weissman JS, Bartel DP (2010) Mammalian microRNAs predominantly act to decrease target mRNA levels. Nature 466: 835–840. 20703300 10.1038/nature09267 PMC2990499

[29] Djuranovic S, Nahvi A, Green R (2011) A parsimonious model for gene regulation by miRNAs. Science 331: 550–553. 21292970 10.1126/science.1191138 PMC3955125

[30] Lim LP, Lau NC, Garrett-Engele P, Grimson A, Schelter JM, et al (2005) Microarray analysis shows that some microRNAs downregulate large numbers of target mRNAs. Nature 433: 769–773. 15685193 10.1038/nature03315

[31] Taganov KD, Boldin MP, Chang KJ, Baltimore D (2006) NF-kappaB-dependent induction of microRNA miR-146, an inhibitor targeted to signaling proteins of innate immune responses. Proc Natl Acad Sci U S A 103: 12481–12486. 16885212 10.1073/pnas.0605298103 PMC1567904

[32] Androulidaki A, Iliopoulos D, Arranz A, Doxaki C, Schworer S, et al (2009) The kinase Akt1 controls macrophage response to lipopolysaccharide by regulating microRNAs. Immunity 31: 220–231. 19699171 10.1016/j.immuni.2009.06.024 PMC2865583

[33] O’Connell RM, Taganov KD, Boldin MP, Cheng G, Baltimore D (2007) MicroRNA-155 is induced during the macrophage inflammatory response. Proc Natl Acad Sci U S A 104: 1604–1609. 17242365 10.1073/pnas.0610731104 PMC1780072

[34] Ruggiero T, Trabucchi M, De Santa F, Zupo S, Harfe BD, et al (2009) LPS induces KH-type splicing regulatory protein-dependent processing of microRNA-155 precursors in macrophages. The FASEB journal : official publication of the Federation of American Societies for Experimental Biology 23: 2898–2908. 19423639 10.1096/fj.09-131342

[35] Tili E, Michaille JJ, Cimino A, Costinean S, Dumitru CD, et al (2007) Modulation of miR-155 and miR-125b levels following lipopolysaccharide/TNF-alpha stimulation and their possible roles in regulating the response to endotoxin shock. J Immunol 179: 5082–5089. 17911593 10.4049/jimmunol.179.8.5082

[36] Tserel L, Runnel T, Kisand K, Pihlap M, Bakhoff L, et al (2011) MicroRNA expression profiles of human blood monocyte-derived dendritic cells and macrophages reveal miR-511 as putative positive regulator of Toll-like receptor 4. J Biol Chem 286: 26487–26495. 21646346 10.1074/jbc.M110.213561 PMC3143613

[37] Lu LF, Boldin MP, Chaudhry A, Lin LL, Taganov KD, et al (2010) Function of miR-146a in controlling Treg cell-mediated regulation of Th1 responses. Cell 142: 914–929. 20850013 10.1016/j.cell.2010.08.012 PMC3049116

[38] Nahid MA, Pauley KM, Satoh M, Chan EK (2009) miR-146a is critical for endotoxin-induced tolerance: IMPLICATION IN INNATE IMMUNITY. J Biol Chem 284: 34590–34599. 19840932 10.1074/jbc.M109.056317 PMC2787321

[39] O’Neill LA, Sheedy FJ, McCoy CE (2011) MicroRNAs: the fine-tuners of Toll-like receptor signalling. Nat Rev Immunol 11: 163–175. 21331081 10.1038/nri2957

[40] Tang Y, Luo X, Cui H, Ni X, Yuan M, et al (2009) MicroRNA-146A contributes to abnormal activation of the type I interferon pathway in human lupus by targeting the key signaling proteins. Arthritis Rheum 60: 1065–1075. 19333922 10.1002/art.24436

[41] Schembri F, Sridhar S, Perdomo C, Gustafson AM, Zhang X, et al (2009) MicroRNAs as modulators of smoking-induced gene expression changes in human airway epithelium. Proc Natl Acad Sci U S A 106: 2319–2324. 19168627 10.1073/pnas.0806383106 PMC2650144

[42] Reisetter AC, Stebounova LV, Baltrusaitis J, Powers L, Gupta A, et al (2011) Induction of inflammasome-dependent pyroptosis by carbon black nanoparticles. J Biol Chem 286: 21844–21852. 21525001 10.1074/jbc.M111.238519 PMC3122239

[43] Monick MM, Carter AB, Gudmundsson G, Geist LJ, Hunninghake GW (1998) Changes in PKC isoforms in human alveolar macrophages compared with blood monocytes. Am J Physiol 275: L389–397. 9700101 10.1152/ajplung.1998.275.2.L389

[44] Monick MM, Powers LS, Barrett CW, Hinde S, Ashare A, et al (2008) Constitutive ERK MAPK activity regulates macrophage ATP production and mitochondrial integrity. J Immunol 180: 7485–7496. 18490749 10.4049/jimmunol.180.11.7485 PMC2410094

[45] Monick MM, Powers LS, Gross TJ, Flaherty DM, Barrett CW, et al (2006) Active ERK Contributes to Protein Translation by Preventing JNK-Dependent Inhibition of Protein Phosphatase 1. J Immunol 177: 1636–1645. 16849472 10.4049/jimmunol.177.3.1636

[46] Monick MM, Powers LS, Walters K, Lovan N, Zhang M, et al (2010) Identification of an autophagy defect in smokers’ alveolar macrophages. J Immunol 185: 5425–5435. 20921532 10.4049/jimmunol.1001603 PMC3057181

[47] Benjamini Y, Krieger A, Yekutieli D (2001) Two staged linear step up FDR controlling procedure. Tel Aviv University, and Department of Statistics, Wharton School, University of Pennsylvania.

[48] Langfelder P, Horvath S (2007) Eigengene networks for studying the relationships between co-expression modules. BMC systems biology 1: 54. 18031580 10.1186/1752-0509-1-54 PMC2267703

[49] Lewis BP, Shih IH, Jones-Rhoades MW, Bartel DP, Burge CB (2003) Prediction of mammalian microRNA targets. Cell 115: 787–798. 14697198 10.1016/s0092-8674(03)01018-3

[50] Griffiths-Jones S, Saini HK, van Dongen S, Enright AJ (2008) miRBase: tools for microRNA genomics. Nucleic Acids Res 36: D154–158. 17991681 10.1093/nar/gkm952 PMC2238936

[51] Tsuchiya S, Kobayashi Y, Goto Y, Okumura H, Nakae S, et al (1982) Induction of maturation in cultured human monocytic leukemia cells by a phorbol diester. Cancer Res 42: 1530–1536. 6949641

[52] Hansdottir S, Monick MM (2011) Vitamin D effects on lung immunity and respiratory diseases. Vitamins and hormones 86: 217–237. 21419273 10.1016/B978-0-12-386960-9.00009-5 PMC3559187

[53] Martinez FO, Gordon S, Locati M, Mantovani A (2006) Transcriptional profiling of the human monocyte-to-macrophage differentiation and polarization: new molecules and patterns of gene expression. Journal of immunology 177: 7303–7311. 10.4049/jimmunol.177.10.7303 17082649

[54] Izzotti A, Calin GA, Arrigo P, Steele VE, Croce CM, et al (2009) Downregulation of microRNA expression in the lungs of rats exposed to cigarette smoke. The FASEB journal : official publication of the Federation of American Societies for Experimental Biology 23: 806–812. 18952709 10.1096/fj.08-121384 PMC2653990

[55] Martinez FO, Sica A, Mantovani A, Locati M (2008) Macrophage activation and polarization. Frontiers in bioscience : a journal and virtual library 13: 453–461. 17981560 10.2741/2692

[56] Stampfli MR, Anderson GP (2009) How cigarette smoke skews immune responses to promote infection, lung disease and cancer. Nature reviews Immunology 9: 377–384. 10.1038/nri2530 19330016

[57] Modestou MA, Manzel LJ, El-Mahdy S, Look DC (2010) Inhibition of IFN-gamma-dependent antiviral airway epithelial defense by cigarette smoke. Respir Res 11: 64. 20504369 10.1186/1465-9921-11-64 PMC2890646

[58] Dhillon NK, Murphy WJ, Filla MB, Crespo AJ, Latham HA, et al (2009) Down modulation of IFN-gamma signaling in alveolar macrophages isolated from smokers. Toxicol Appl Pharmacol 237: 22–28. 19269302 10.1016/j.taap.2009.02.021 PMC2680937

[59] Pottelberge GR, Mestdagh P, Bracke KR, Thas O, Durme YM, et al (2011) MicroRNA expression in induced sputum of smokers and patients with chronic obstructive pulmonary disease. Am J Respir Crit Care Med 183: 898–906. 21037022 10.1164/rccm.201002-0304OC

[60] D’Hulst A I, Bracke KR, Maes T, De Bleecker JL, Pauwels RA, et al (2006) Role of tumour necrosis factor-alpha receptor p75 in cigarette smoke-induced pulmonary inflammation and emphysema. The European respiratory journal : official journal of the European Society for Clinical Respiratory Physiology 28: 102–112. 10.1183/09031936.06.00059305 16540505

[61] Gao C, Guo H, Mi Z, Grusby MJ, Kuo PC (2007) Osteopontin induces ubiquitin-dependent degradation of STAT1 in RAW264.7 murine macrophages. Journal of immunology 178: 1870–1881. 10.4049/jimmunol.178.3.1870 17237438

[62] Guo H, Wai PY, Mi Z, Gao C, Zhang J, et al (2008) Osteopontin mediates Stat1 degradation to inhibit iNOS transcription in a cecal ligation and puncture model of sepsis. Surgery 144: 182–188. 18656624 10.1016/j.surg.2008.03.007 PMC2525867

[63] Gaur A, Jewell DA, Liang Y, Ridzon D, Moore JH, et al (2007) Characterization of microRNA expression levels and their biological correlates in human cancer cell lines. Cancer Res 67: 2456–2468. 17363563 10.1158/0008-5472.CAN-06-2698

[64] Lu J, Getz G, Miska EA, Alvarez-Saavedra E, Lamb J, et al (2005) MicroRNA expression profiles classify human cancers. Nature 435: 834–838. 15944708 10.1038/nature03702

[65] Thomson JM, Newman M, Parker JS, Morin-Kensicki EM, Wright T, et al (2006) Extensive post-transcriptional regulation of microRNAs and its implications for cancer. Genes & development 20: 2202–2207. 16882971 10.1101/gad.1444406 PMC1553203

[66] Kumar MS, Lu J, Mercer KL, Golub TR, Jacks T (2007) Impaired microRNA processing enhances cellular transformation and tumorigenesis. Nat Genet 39: 673–677. 17401365 10.1038/ng2003

